# Patient-reported outcomes measure for patients with cleft palate

**DOI:** 10.3389/fpubh.2024.1469455

**Published:** 2024-08-29

**Authors:** Wenbo Xia, Meijun Du, Min Wu, Zehua Chen, Renjie Yang, Bing Shi, Hanyao Huang

**Affiliations:** ^1^State Key Laboratory of Oral Diseases and National Clinical Research Center for Oral Diseases and Department of Oral and Maxillofacial Surgery, West China Hospital of Stomatology, Sichuan University, Chengdu, China; ^2^State Key Laboratory of Oral Diseases and National Clinical Research Center for Oral Diseases and Eastern Clinic, West China Hospital of Stomatology, Sichuan University, Chengdu, China

**Keywords:** quality of life, cleft palate, patient-reported outcomes measure, velopharyngeal insufficiency, patient perception

## Abstract

Cleft palate presents multifaceted challenges impacting speech, hearing, appearance, and cognition, significantly affecting patients’ quality of life (QoL). While surgical advancements aim to restore function and improve appearance, traditional clinical measures often fail to comprehensively capture patients’ experiences. Patient-reported outcomes measure (PROMs) have emerged as crucial tools in evaluating QoL, offering insights into various aspects such as esthetic results, speech function, and social integration. This review explores PROMs relevant to cleft palate complications, including velopharyngeal insufficiency, oronasal fistulas, maxillary hypoplasia, sleep-disordered breathing, and caregiver QoL. Additionally, the review highlights the need for cleft palate-specific scales to better address the unique challenges faced by patients. By incorporating PROMs, healthcare providers can achieve more personalized, patient-centered care, improve communication, and enhance treatment outcomes. Future research should focus on developing and validating specialized PROMs to further refine patient assessments and care strategies.

## 1 Introduction

Cleft palate may affect the soft/hard palate and alveolar region (1), significantly impacting speech, hearing, appearance, and cognition (2). Surgical techniques have been developed to restore velopharyngeal function and normal appearance, aiming to improve quality of life (QoL). However, traditional objective measures of surgical outcomes often lack the comprehensiveness needed to convey patients’ experiences to clinicians. Patient-reported outcomes measures (PROMs) are employed to gather information on patients’ QoL. PROMs offer a wide range of insights, including esthetic results, speech function, self-image, social integration, etc., obtained directly from simply one patient-completed questionnaire (3).

Cleft palate may significantly impact patients throughout their lives. Early challenges, such as feeding difficulties, can affect the patient’s growth (4). Meanwhile, cleft palate increases the risk of ear infections and hearing loss since eustachian tubes are poorly developed and food can easily enter the ear (5, 6). What’s more, speech dysfunctions and altered appearance can lead to social struggle due to difficulty in communication and odd pronunciation (7). Incorrect surgical treatments (e.g., bad timing or wrong technique) may result in complications such as oronasal fistulas, which allows food and liquids to enter the nasal cavity, inducing inflammation and halitosis (7). Sleep problems are also common after cleft palate repair, especially following secondary velopharyngeal insufficiency (VPI) correction, and sleep deprivation can hinder growth, cause learning difficulties, and delay socialization (8). The bond between children with cleft palate and their caregivers is crucial for their self-esteem and development (9). Overall, cleft palate presents complex challenges to the patient’s entire life growth that are difficult to fully resolve through treatment and affects the patient’s QoL.

Therefore, it is paramount to utilizing appropriate instruments to comprehensively understand the nuanced aspects of the patient’s QoL. General questionnaires, like the Child Oral Health Impact Profile (COHIP) and the Child Oral Health Quality of Life Questionnaire (COHQOL), have been developed to measure the overall quality of life in children with cleft palate. However, the lack of scales targeting specific issues related to cleft palate makes it challenging for clinicians and researchers to obtain detailed information. There are generic questionnaires to measure the QoL in specific symptoms related to cleft palate and cleft palate-related complications (10). Although they may not be highly relevant to cleft palate, they can still collect more detailed data in specific areas. Herein, this mini review aims to give a brief introduction to these various assessment tools for cleft-palate-complications-related PROMs, which may help in developing cleft-palate-specific scales in the future.

## 2 Complications after cleft palate repair and patient-reported outcomes measure

### 2.1 Velopharyngeal insufficiency

VPI can cause hypernasality and nasal air emission, leading to incomprehensible speech and affecting psychological well-being (11, 12). For PROMs related to VPI, we can use either questionnaires specifically designed for VPI or those focused on general speech-related issues (Table 1, Part 1).

**Table 1:** Comparison of different PROMs for velopharyngeal insufficiency and speech, and oronasal fistula.

Instrument	Target	Items	Domains	Development year	Response options
**Part 1. Comparison of different PROMs for velopharyngeal insufficiency and speech**					
VPIQL	Determine changes of QoL in children with VPI between 5 and 17 years old	48	Speech limitations, swallowing problems, situational difficulty, emotional impact, perception by others, activity limitations and caregiver impact	2007	5-point Likert type
VELO	Measure QoL in patients with VPI	26	Speech limitation, swallowing problems, situational difficulty, emotional impact, perception by others, caregiver impact	2012	5-point Likert type
PVRQOL	Measure voice changes in pediatric population	10	NA^a^	2006	6-point Likert type
VHI	Calculate the psychosocial adverse effects of voice abnormalities	30	Emotional, functional, physical	1997	5-point Likert type
VHI-9i	Calculate the psychosocial adverse effects of voice abnormalities with short form	9	Emotional, functional, physical	2009	5-point Likert type
SWALQOL	Measure patient-based, dysphagia-specific outcomes	44	Burden, eating duration, eating desire, symptom frequency, food selection, communication, fear, mental health, social, fatigue, sleep	2000	5-point Likert type
**Part 2. Comparison of different PROMs for oronasal fistula**					
RQLQ	Measure QoL in rhinoconjuncivitis for clinical trials	28	Systemic symptoms, sleep disturbance, practical problems, activity limitations, emotional problems	1991	4-point Likert type
mini-RQLQ	Simplify RQLQ	14	Activity limitations, practical problems, nose symptoms, eye symptoms, other symptoms	2000	7-point Likert type
NOSE	Perform a prospective assessment of subjective treatment outcomes	5	NA^a^	2004	4-point Likert type
HALT	Monitor patient’s treatment progress	20	NA^a^	2011	5-point Likert type
VHI-9i	Calculate the psychosocial adverse effects of voice abnormalities with short form	9	Emotional, functional, physical	2009	5-point Likert type
Halfins	Serve as a tool for everyday practice that measures halitosis	15	NA^a^	2021	4-point Likert type
EAT-10	Serve as a rapid tool scoring symptom severity, quality of life, and treatment efficacy in clinical condition	10	NA^a^	2008	5-point Likert type
MCH-FS	Serve as a solid and trustworthy tool that can immediately confirm concerns from parents regarding their child’s eating problems	14	Oral motor, oral sensory, appetite, maternal concerns about feeding, mealtime behaviors, maternal strategies used, family reactions to their child’s feeding	2011	7-point Likert type

a NA, not available, the questionnaire has no subdomain.

The Velopharyngeal Insufficiency Quality of Life instrument (VPIQL) measures VPI impact in US patients (13), yet its length can be cumbersome for clinical use. Its revised version, VPI Effects on Life Outcomes (VELO), reduces patient and caregiver burden and is used in multiple countries, including China (14, 15), Nepal (16), Spain (17, 18), the Netherlands (19), Brazil (20), etc. It serves as a simple tool to help clinicians understand the social, emotional, and physical influences of VPI (14).

The Voice-Related Quality of Life Measure (VRQOL) is for adults, but does not fit for children (21). Its’ child-adapted version, the Pediatric Voice-Related Quality of Life survey (PVRQOL), provides a comprehensive view of children’s issues but lacks direct patient feedback (22). PVRQOL is more detailed than the Pediatric Voice Outcomes Survey (PVOS), though PVOS’s length limits its subdomain specificity (23), indicating its potential to measure voice-related quality of life (24).

The above questionnaires measure children’s overall quality of life rather than specific aspects. To measure psychosocial aspects, researchers and clinicians can consider the 9-Item Voice Handicap Index (VHI-9i), adapted from the Voice Handicap Index (VHI) (25). In clinical experience, VHI-9i is time-saving and patient-friendly, increasing its acceptance and practicality (25, 26).

Although the Swallowing Quality of Life questionnaire (SWAL-QOL) mainly focuses on evaluating chewing function, it can also assess communication-related QoL issues with oropharyngeal dysphagia (27). It can be a reference when designing instruments for cleft palate.

### 2.2 Oronasal fistula

Patients with ONF may experience sinusitis, food impaction, and halitosis due to food entering the nasal cavity through the fistula, as well as speech dysfunction related to the fistula itself (28). PROMs related to ONF can be evaluated using questionnaires designed for nasal functions, speech, and feeding-related issues (Table 1, Part 2).

The Rhino conjunctivitis Quality of Life Questionnaire (RQLQ) has been shortened to mini-RQLQ (29) for efficiency in large clinical trials and practice monitoring. It is reliable for patients with stable rhino conjunctivitis between clinic visits (30). While mini-RQLQ has strong measurement properties, its usefulness in patients with cleft palate needs further investigation.

It is reported that ONF can lead to nasal obstruction (31). Thus, the Nasal Obstruction Symptom Evaluation (NOSE) is a useful global tool for evaluating patients’ nose obstruction symptoms, correlating well with examination findings (32, 33). Halitosis, causing social discomfort, can be measured by the Halitosis Associated Life-quality Test (HALT), which monitors disease progression. The scale’s goal is to potentially measure change rather than draw conclusions about therapy effectiveness (34). The cut point is 14 and higher indicates halitosis. However, these should be used with organoleptic tests for a comprehensive diagnosis in all cases (35).

Speech impairment related to cleft palate can be assessed using VPIQL, VELO, PVOS, PVRQOL, VHI, and VHL-9i presented, as previously mentioned. VELO also evaluates swallowing problems in ONF patients, though it includes non-specific scales. Eating issues are significant in children with ONF, addressed by the 10-item Eating Assessment Tool (EAT-10), which monitors dysphagia severity and treatment efficacy (36). The Montreal Children’s Hospital Feeding Scale (MCH-FS) addresses parental concerns about feeding problems, which may otherwise be overlooked in clinical conditions, with a bilingual format and clinical significance indicators (37).

Other pediatric feeding assessments include Behavioral assessment scale of oral functions in feeding (BASOFF) (38), Dysphagia Disorders Survey (DDS) (39), Functional Feeding Assessment modified (FFAm) (40), Standardized Eating Assessment (GVA) (40), Oral Motor Assessment Scale (OMAS) (41), Pediatric Assessment Scale for Severe Feeding Problems (PASSFP) (42), (Schedule for Oral-Motor Assessment) SOMA (43), and Screening Tool of Feeding Problems applied to children (STEP-CHILD) (44). However, many of these scales are too disease-specific for patients with cleft palate or do not align with their feeding behaviors. Despite this, they still hold potential for use in special conditions (45).

### 2.3 Maxillary hypoplasia

In individuals with cleft palate, the midfacial growth is often disrupted leading to maxillary hypoplasia. This means the maxilla does not develop to its full size and may appear smaller than normal. Additionally, cleft palate can cause malocclusion and other dental issues. Improper orthodontic treatment can also result in maxillary hypoplasia. This condition not only causes dental problems but can also lead to respiratory issues, speech impairment, facial asymmetry, esthetic concerns, and difficulties in chewing. To assess the overall impact of maxillary hypoplasia, several oral health-related questionnaires can be applied (Table 2, Part 1).

**Table 2:** Comparison of different PROMs for maxillary hypoplasia, sleeping-disordered breath, and caregiver.

Instrument	Target	Items	Domains	Development year	Response options
**Part 1. Comparison of different PROMs for maxillary hypoplasia**					
COHIP	Assess oral-facial well-being in school-age children	34	Functional well-being, psychological well-being, social well-being, school, self-image	2007	5-point Likert type
COHQOL (CPQ)	Measure self-reported oral health–related quality of life in children aged 11 to 14 years	37 (default) 8/16 (short form)	Oral symptoms, functional limitations, emotional well-being, social well-being (original form)	2002	5-point Likert type
CLEFT-Q	Measure outcomes that matter to children and young adults with CL/P	/	Appearance: cleft lip scar, face, jaws, nose, nostrils, teeth facial function, eating & drinking, speech health-related quality of life: psychological, school, social, speech distress	2017	4-point Likert type
FaCE Scale	Measure both facial impairment and disability	15	Facial movement, facial comfort, oral function, eye comfort, lacrimal control, social function	2001	5-point Likert type
FDI	Measuring the disability associated with facial nerve disorders exists	10	Physical function, social/well-being function	1996	6-point type
YQOL-FD	Involve patients in comparing treatment effects	48	Stigma, negative self-image, positive consequences, negative consequences, coping	2007	10-point type, 0 = not at all to 10 = a great deal or completely
**Part 2. Comparison of different PROMs for sleeping-disordered breath**					
BQ	Identify patients with sleep apnea in primary care settings	10	Snoring behavior, waketime sleepiness or fatigue, the presence of obesity or hypertension	1999	Frequency (Almost every day, 3–4 times/wk., 1–2 times/wk., 1–2 times/mo., Never or almost never), Tendency (Increased, Decreased, No change), Yes/No/Do not know
ESS	Measure sleep propensity in a simple, standardized way	8	NA^a^	1991	4-point Likert type
SBQ	Serve as a concise and easy-to-use questionnaire for OSA screening in surgical patients	8	Snoring, tiredness, observed apnea, high blood pressure, BMI, age, neck circumference, male gender	2008	Yes/No, Yes = 1, No = 0 m
WUSQ	Address public impart of sleeping-disordered breath	32	Snoring and sleep-disordered breathing, disturbed sleep, personal and family medical history, life habits	1992	5-point frequency scale + “I do not know” category
PSQI	(1) Provide a reliable stable, credible, standardized measure of sleeping quality (2) Sort out the good sleepers from the bad ones (3) Provide a user-friendly scale for patients, clinicians, and researchers (4) Provide a tool assessing sleep disruptions that impair sleep quality	19	Subjective sleep quality, sleep latency, sleep duration, habitual, sleep efficiency, sleep disturbances, use of sleeping medications, daytime dysfunction	1988	0–3 component score depending on frequency/duration/feeling/component score/
FOSQ		30	Activity level, vigilance, intimacy and sexual relationships, general productivity, social outcome	1997	5-point scale
CSHQ		33	bedtime resistance, sleep onset delay, sleep duration, sleep anxiety, night waking, parasomnias, sleep-disordered breathing, daytime sleepiness	2002	3-point scale
PSQ	Investigate the presence of childhood SRBDs and prominent symptom complexes, including snoring, daytime sleepiness, and related behavioral disturbances	22	Snoring, sleepiness, behavior	2000	“Do not apply” and “apply just a little” are scored as no (0), “apply quite a bit” and “definitely applies most of the time” were scored as yes
**Part 3. Comparison of different PROMs for caregiver**					
CSI	Identify families with potential caregiving issues	13	NA^a^	1983	Yes/No, make examples
CBI	Assess caregiver burden impact	24	Time-dependence burden, developmental burden, physical burden, social burden, e motional burden	1989	5-point Likert type
CarerQoL	Measure caregiver burden	7 + 1 (VAS)	Care-related fulfillment, social support, relationship issues with the care recipient, financial security, challenges finishing daily tasks, physical health issues	2006	No/Some/A lot of + VAS
PedsQL Family Impact Module	Assess pediatric QoL to assess risk and track health status	45	Physical functioning, psychological functioning, social functioning	1999	4-point Likert type
C-FRAS	Measure family resilience	44 (default) 16 (short form)	Family communication and problem solving, utilizing social and economic resources, maintaining a positive outlook, ability to make meaning of adversity	2022	4-point Likert type
IOFS	Quantify the impact of childhood illness on a family	24	Financial load, social contacts both inside and outside home, parent’s personal strain or discomfort, feeling of loss of control brought on by stress	1980	4-point Likert type
FIS	Assess the impact of children’s oral problems to family	14 (default) 8 (short-form)	Orofacial disease impact, parental emotion, family conflict, family economic	2002	4-point Likert type

a NA, not available, the questionnaire has no subdomain.

Child’s Oral Health Impact Profile (COHIP) is a well-designed tool that has shown excellent reliability in measuring oral-facial well-being among children aged 8–15. It assesses several significant issues in patients with cleft palate and is cleft palate specific. The questionnaire can distinguish differences in oral health-related quality of life between those with craniofacial anomalies and those without (46, 47).

Child Oral Health Quality of Life Questionnaire (COHQOL) consists two components: the Parent-Caregiver Perception Questionnaire (P-CPQ) and the Child Perceptions Questionnaire (CPQ). The CPQ is a self-administered tool measuring oral health-related quality of life in children aged 11–14, originally containing 37 items, with 16-item and 8-item shortened versions developed for clinical use (48–50).

CLEFT-Q, a rigorously developed instrument, includes a series of scales across three domains with 12 minor themes, suitable for patients aged 8 to 29 years. Each scale can be used separately to reduce patient burden, as patients only complete scales relevant to their problems. There is no total score, making it flexible for addressing specific concerns such as appearance, facial function, and health-related quality of life (51–53).

Facial Clinimetric Evaluation Scale (FaCE Scale) is originally developed for evaluating facial paralysis but has been adapted to assess facial dysfunction, a key feature of maxillary hypoplasia. It provides scores across seven areas: facial movement, facial comfort, oral function, eye comfort, lacrimal control, social function, and total score (54).

Facial Disability Index (FDI), a disease-specific instrument, examines physical impairment and psychosocial variables in individuals with facial nerve disorders. It features two domains, each with five items, and domain scores are transformed to a 100-point scale. Its brevity allows for quick completion and immediate score comparison, fitting well in outpatient settings (55).

The Youth Quality of Life-Facial Differences Questionnaire (YQOL-FD) is a craniofacial-specific quality of life module that complements the generic Youth Quality of Life Instrument (YQOL). It is suitable for youth aged 11–18 and readable for children in the fifth grade. The tool highlights the impact of facial differences on QoL from the patient’s perspective, offering a patient-centered profile for comparing treatment effects beyond clinician-derived outcomes of esthetics and function (56). However, it is not specifically customized for patients with cleft palate and may overlook some crucial aspects significant to this population (3).

### 2.4 Sleep-disordered breath

Patients with cleft palate are more susceptible to SDB. The cleft in the roof of the oral cavity can result in smaller airways and an abnormal nasal cavity, leading to breathing difficulties during sleep. Additionally, surgical repair of the cleft palate may contribute to SDB due to scarring and changes in the shape and function of the palate and surrounding tissues, which can further narrow the airway. Common symptoms of SDB in individuals with cleft palate include loud snoring, gasping or choking during sleep, restless sleep, and daytime sleepiness. The most prevalent condition among these patients is Obstructive Sleep Apnea (OSA), characterized by repeated episodes of partial or complete upper airway collapse during sleep, leading to decreased oxygen levels and disrupted sleep. If left untreated, SDB in individuals with cleft palate can lead to long-term complications such as growth and developmental problems, cognitive deficits, and cardiovascular diseases. Therefore, applying assessment scales to guide timely treatment is essential (Table 2, Part 2).

The Berlin Questionnaire (BQ) identifies patients at risk of sleep apnea by asking risk factors. A patient is considered at high risk for sleep apnea if they exhibit symptoms in at least two categories. Those qualified for only one symptom category are considered as lower risk (57). The Epworth Sleepiness Scale (ESS) measures daytime sleepiness, with scores above 16 indicating a high tendency for daytime drowsiness (58). The STOP questionnaire is a brief and simple OSA screening tool for surgical patients, focusing on Snoring, Tiredness, Observed apnea, and high blood Pressure. The STOP-Bang questionnaire (SBQ) includes four additional demographic questions (BMI, Age, Neck circumference, Male gender), enhancing its sensitivity and effectiveness (59, 60). A score of 0–2 on the SBQ implies low risk of OSA, while a score of 3 or more indicates a higher risk. The SBQ is a more reliable instrument for identifying mild, moderate, and severe OSA than the BQ, STOP, and ESS (61).

The Wisconsin University Sleep Questionnaire (WUSQ), adapted from the Basic Northern Sleep Questionnaire, has been translated and validated for consistency (62, 63). The Pittsburgh Sleep Quality Index (PSQI) assesses sleep quality over the previous month, distinguishing transient disturbances from persistent ones. It provides a “global” score indicating the severity of sleep difficulties (64). The Functional Outcomes of Sleep Questionnaire (FOSQ) measures how sleepiness affects daily functioning and is suitable for children above the 5th grade. Though designed to evaluate disorders of excessive sleepiness (DOES), it can also measure SDB-related quality of life (65).

Given that most cleft palate patients are children, pediatric-specific scales are necessary. The Children’s Sleep Habits Questionnaire (CSHQ) is a parent-report tool diagnosing sleep disorders in school-aged children (4–10) based on a typical week’s sleep behavior (66). The Pediatric Sleep Questionnaire (PSQ) investigates childhood sleep-related breathing disorders and symptoms like snoring and daytime sleepiness in children aged 2–18 years, making it useful in clinical research when polysomnography is unavailable (67).

### 2.5 Caregiver QoL

Caregivers’ attitudes influence patients’ recovery, while caregivers’ feelings toward their child’s cleft defect also play a crucial role in the development of the child’s self-esteem (9). While the response of caregivers to their child with cleft palate is well-documented (68), the impact of caregivers’ psychological status on their children needs further study by PROMs (Table 2, Part 3).

The Caregiver Strain Index (CSI) identifies families with potential caregiving issues, with a score above 6 indicating the need for further assessment. However, CSI is designed for patients over 65 and lacks a subjective assessment of caregiving impact (69). The Caregiver Burden Inventory (CBI) is a multidimensional tool assessing caregiver burden, but dose not account for its impact on caregiver QoL.

The Care-Related Quality of Life Instrument (CarerQoL) includes two scales: CarerQoL-7D, which measures caregiver burden in two positive dimensions and five negative dimensions (70), and CarerQoL-VAS, which determines patients’ happiness by drawing a X on a number axis (71). CarerQoL-7D is widely used for informal caregivers, reflecting the situation of parents with children with cleft palate (72–75).

Caregiver stress can negatively impact families, causing emotional pressure, relationship strain, and health problems. Thus, assessing family impact of caregiver pressure is significant. The PedsQL Family Impact Module, part of the PedsQL measure, assesses parent self-reported QoL to evaluate risk and track health status (76). Parents’ perceptions of their children’s QoL often reflect their own stress levels. The Chinese Family Resilience Assessment Scale (C-FRAS) measures family resilience, with higher scores indicating greater resilience (77), and can be used to assess the resilience of families with a child who has a cleft palate.

The Impact on Family Scale (IOFS) quantifies the impact of childhood illness on families, with higher scores indicating a greater detrimental effect (78). IOFS reliably detects changes, making it a valuable tool for monitoring a family during illness course (79). The scale has been applied in monitoring the family impact of childhood cancer (80) and children with posterior urethral valves (81), obstetrical brachial plexus injury (82) and cleft palate (83). It confirms that having a child with cleft palate affects parents’ QoL (83). The Family Impact Scale (FIS), part of the Parental-Caregivers Perceptions Questionnaire (P-CPQ) (84), quantifies the impact of a child’s oral problems on the family. FIS is valid in determining the impact of orofacial cleft on family QoL (85), and its short form, FIS-8, has shown great internal consistency reliability (86). However, its sociodemographic patterns in China need further research.

## 3 Conclusion

Compared to traditional doctor-guided evaluation methods, PROMs better capture patients’ perspectives, especially their perceptions of their own health and QoL. PROMs serve as supplementary tools for assessing treatment effectiveness, aiding in clinical decision-making. Their application leads to more personalized care, allowing healthcare providers to tailor treatments to individual patient needs and preferences. PROMs facilitate better communication between patients and healthcare providers by promoting discussions about patient experiences, concerns, and treatment goals, significantly improving patient satisfaction. Medical institutions can assess healthcare quality by tracking PROMs over time.

In summary, PROMs are crucial for patients with cleft palate as they provide unique insights into the patient’s perspective, inform treatment decisions, enhance communication, evaluate healthcare quality, and support research. By applying PROMs, healthcare providers ensure comprehensive, patient-centered care for individuals with cleft palate.
